# Voltage sensor dynamics of a bacterial voltage-gated sodium channel NavAb reveal three conformational states

**DOI:** 10.1016/j.jbc.2023.102967

**Published:** 2023-02-01

**Authors:** Shuo Han, Joshua Vance, Samuel Jones, Jenna DeCata, Kimberly Tran, John Cummings, Shizhen Wang

**Affiliations:** Division of Biological and Biomedical Systems, School of Science and Engineering, University of Missouri-Kansas City, Kansas City, Missouri, USA

**Keywords:** voltage-gated ion channels, ion channels, single-molecule FRET, structural dynamics, voltage sensor, ACMA, 9-amino-6-chloro-2-methoxyacridine, AEBSF, 4-benzenesulfonyl fluoride hydrochloride, CCCP, carbonyl cyanide m-chlorophenyl hydrazone, Hepes, 2-[4-(2-hydroxyethyl)piperazin-1-yl]ethanesulfonic acid, LMNG, lauryl maltose neopentyl glycol, NMDG, N-methyl-D-glucamine, smFRET, single-molecule fluorescence resonance energy transfer, Tris, tris(hydroxymethyl)aminomethane, Trolox, 6-hydroxy-2,5,7,8-tetramethylchroman-2-carboxylic acid, VGIC, voltage-gated ion channel

## Abstract

High-resolution structures of voltage-gated sodium channels (Nav) were first obtained from a prokaryotic ortholog NavAb, which provided important mechanistic insights into Na^+^ selectivity and voltage gating. Unlike eukaryotic Navs, the NavAb channel is formed by four identical subunits, but its ion selectivity and pharmacological profiles are very similar to eukaryotic Navs. Recently, the structures of the NavAb voltage sensor at resting and activated states were obtained by cryo-EM, but its intermediate states and transition dynamics remain unclear. In the present work, we used liposome flux assays to show that purified NavAb proteins were functional to conduct both H^+^ and Na^+^ and were blocked by the local anesthetic lidocaine. Additionally, we examined the real-time conformational dynamics of the NavAb voltage sensor using single-molecule FRET. Our single-molecule FRET measurements on the tandem NavAb channel labeled with Cy3/5 FRET fluorophore pair revealed spontaneous transitions of the NavAb S4 segment among three conformational states, which fitted well with the kinetic model developed for the S4 segment of the human voltage-gated proton channel hHv1. Interestingly, even under strong activating voltage, the NavAb S4 segment seems to adopt a conformational distribution similar to that of the hHv1 S4 segment at a deep resting state. The conformational behaviors of the NavAb voltage sensor under different voltages need to be further examined to understand the mechanisms of voltage sensing and gating in the canonical voltage-gated ion channel superfamily.

Voltage-gated ion channels (VGICs) conduct ions across the membrane barrier in response to membrane voltage. Inherited dysfunctional mutations of VGICs are associated with cardiac, muscular, neurological, and psychiatric disorders such as long, short Q.T. syndrome, periodic paralysis, epilepsy, bipolar disorder, etc. (1, 2). Eukaryotic voltage-gated sodium (Nav) and calcium (Cav) channels are formed by a single pore-forming subunit with four homologous domains, and each domain has six transmembrane segments, named S1 through S6 (3). Among them, S1-S4 segments form voltage sensors, with the S4 containing several charged residues to sense membrane voltage (4). The crystal structures of Navs were first obtained from prokaryotic homologs NavAb, for it is a homotetrameric Nav channel with superior yield, stability, and homogeneity than its eukaryotic orthologs (5, 6). The NavAb channel shares high structural similarities to eukaryotic Nav/Cav channels (7, 8, 9, 10, 11). As a homotetrameric channel, although NavAb lacks the asymmetric ‘DEKA’ signature sequences of eukaryotic Nav channels (4), its selectivity filter aligns very well with those of eukaryotic orthologs (5, 8, 9, 12). More importantly, the ion selectivity and pharmacological profiles of the NavAb channel are also very similar to these of eukaryotic Navs (13, 14). Since its atomic structure was solved in 2011, the NavAb channel has been a model for investigating the mechanisms of ion permeation and voltage gating. Recently, the structures of both resting and activated NavAb channels were obtained by cryo-EM using a cysteine cross-linking strategy, which indicated the voltage sensing S4 segment moves ∼11 Å and translocates three gating charges across the hydrophobic constriction site during gating transition (15). Similar changes were also found on voltage sensors from two-pore channels TPC1 (16, 17). However, all these observed conformational changes at the S4 segment in voltage sensors were much less than the movements of three or even four helical turns (*i.e.*, 15 ∼ 20 Å) suggested by electrophysiological studies (18, 19).

In the present work, we purified NavAb channels and reconstituted them into liposomes. For the first time, we examined the function of purified NavAb proteins by liposome flux assay and showed that they are highly permeable to both H^+^ and Na^+^, which can be blocked by lidocaine. We also constructed a functional tandem tetrameric NavAb channel by concatenating four NavAb subunits and introduced a pair of cysteine residues to only one of the four voltage sensors. We performed single-molecule FRET (smFRET) measurements on purified tandem NavAb channels labeled with Cy3/Cy5 maleimide fluorophore pair, which uncovered spontaneous movements of the S4 segment in the NavAb voltage sensor. Our smFRET results indicated that the S4 segment in the NavAb voltage sensor has a conformational landscape similar to that of the hHv1 channel, a structural basis for them to share similar mechanisms in sensing membrane voltage.

## Results

### Purified NavAb channels conduct both H^+^ and Na^+^

**Figure 1:**
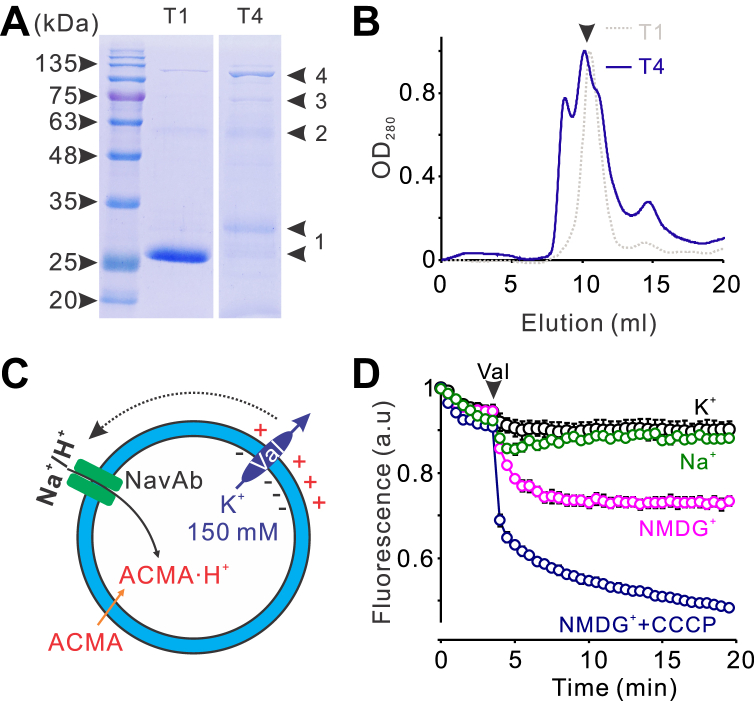
**Functional characterizations of purified NavAb channel proteins.***A*, SDS-PAGE of the monomeric (T1) and tandem tetrameric (T4) NavAb proteins purified by metal affinity chromatography. *B*, gel filtration profile of the affinity-purified NavAb proteins. *C*, liposome flux assay to examine the function of the NavAb channel proteins reconstituted into liposomes. *D*, liposome flux assay data of NavAb proteoliposomes. The intraliposomal buffer containing 20 mM Hepes, 150 KCl, pH7.5 and the extraliposomal buffer containing 150 KCl (K^+^), 150 mM NaCl (Na^+^), or 150 mM NMDG without (NMDG^+^) or with 1 μM CCCP (NMDG^+^+CCCP). The assays were started by adding 0.45 μM valinomycin (marked by arrow). All data were presented as mean±s.e, n = 3. Hepes, 2-[4-(2-hydroxyethyl)piperazin-1-yl]ethanesulfonic acid; NMDG, N-methyl-D-glucamine; CCCP, carbonyl cyanide m-chlorophenyl hydrazone.

The function of the NavAb channel, including its pharmacological profiles, has been extensively characterized by patch-clamp electrophysiology on those expressed in eukaryotic cells (5, 13, 20). Surprisingly, the function of purified NavAb proteins for structural determinations was never examined. We successfully expressed and purified the NavAb-N49K/T206A mutant in *Escherichia coli* KRX host cells (Fig. 1, *A* and *B*) and then reconstituted NavAb proteins into liposomes (1-palmitoyl-2-oleoyl-sn-glycero-3-phosphoethanolamine/1-palmitoyl-2-oleoyl-sn-glycero-3-phospho-(1'-rac-glycerol) = 3/1). The activities of purified NavAb proteins were examined by liposome flux assay (Fig. 1*C*), where transliposomal K^+^ gradient, in the presence of K^+^ ionophore valinomycin, generated electrical potentials to drive the Na^+^ or H^+^ uptake into liposomes. H^+^ uptake into liposomes is reported by quenching of the pH-sensitive fluorophore 9-amino-6-chloro-2-methoxyacridine (ACMA). As shown in Figure 1*D*, although less potent than proton ionophore CCCP, robust ACMA quenching was observed in the presence but not in the absence of the K^+^ gradient after adding valinomycin, indicating that NavAb can mediate the uptake of H^+^ into liposomes. The ACMA quenching is abolished by external Na^+^, suggesting the NavAb channel is also permeable to Na^+^, leading to dissipation of K^+^ potential, thus suppressing H^+^ uptake into liposomes. We also noticed a slow quenching of ACMA fluorescence before adding valinomycin, which agrees with the fact that NavAb channels are also permeable to K^+^, although only less than 20% in comparison to Na^+^ (5). Moreover, with K^+^ potential at more acidic extraliposomal pH, we observed slightly more ACMA quenching (Fig. S1*A*); with H^+^ gradient without K^+^ potential, we observed the recovery of ACMA quenching as the result of dissipating H^+^ gradient by NavAb channels (Fig. S1*B*). In summary, our liposome flux data indicated that purified NavAb proteins are functional to conduct both Na^+^ and H^+^, consistent with the ion selectivity profiles of NavAb and other Navs determined by electrophysiology (4, 5).

**Figure 2:**
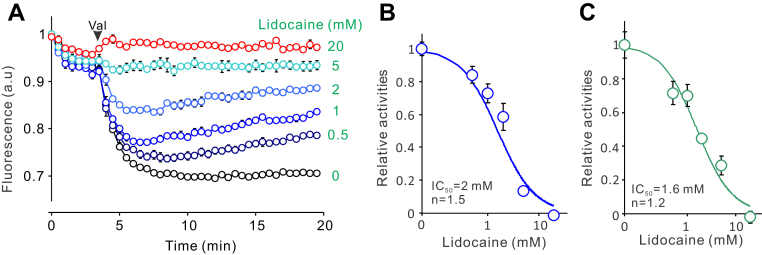
**Lidocaine blocks the NavAb channel in a dose-dependent manner.***A*, liposome flux assay data of monomeric tetrameric NavAb proteoliposomes at different concentrations of lidocaine added in extraliposomal buffer. *B* and *C*, dose-dependent blocking of the monomeric (*B*) and tandem tetrameric (*C*) NavAb channels by lidocaine, fitted with the Hill equation. All data were presented as mean±s.e, n = 3.

The NavAb channel is also blocked by local anesthetics and antiarrhythmic drugs like lidocaine and flecainide-targeting eukaryotic Navs (13). As shown in Figure 2*A*, the purified NavAb channels reconstituted into liposomes also exhibited dose-dependent blocking by lidocaine. Fitting the blocking curve with the Hill equation yielded a half-blocking concentration of 2 mM, very close to 2.4 mM, obtained by patch-clamp studies on the NavAb channel expressed in insect cells (13). It is worth noting that these assays were performed in the absence of proton ionophore CCCP, which suggested that lidocaine blocks both Na^+^ and H^+^ uptakes.

### Tandem tetrameric NavAb channels are functional

The NavAb channel is free of intrinsic cysteine residues, making it an ideal candidate for studying conformational dynamics using the smFRET approach. However, to label the tetrameric channel unit with a single FRET fluorophore pair, a tandem channel carrying only two introduced cysteine residues is required, which we previously achieved on a tetrameric potassium channel (21, 22). We prepared a tandem construct by linking four NavAb cDNAs with linkers encoding the flexible 2×’GGGS’ and 1× thrombin cutting site. The construct was successfully expressed and purified following the same protocol as the monomeric NavAb channel, although the yield dropped by ∼5 folds to 0.05 mg per liter of culture. Unlike the monomeric NavAb channel, the tandem tetrameric NavAb channel appeared primarily as tetrameric bands in SDS-PAGE, with some minor trimer, dimer, and monomer bands, perhaps resulting from linker degradation during the purification (Fig. 1*A*). But, the tandem tetrameric NavAb proteins are less conformationally homogeneous than the monomeric NavAb protein, so only the tetrameric peak fractions were collected for structural and functional characterizations (Fig. 1*B*). Liposome flux assay data indicated that the tandem tetrameric NavAb channel is also functional and sensitive to lidocaine block, with a half-blocking concentration of 1.6 mM, very close to the monomeric NavAb channel (Fig. 2*C*).

### The conformational dynamics of the NavAb voltage sensor revealed by smFRET

**Figure 3:**
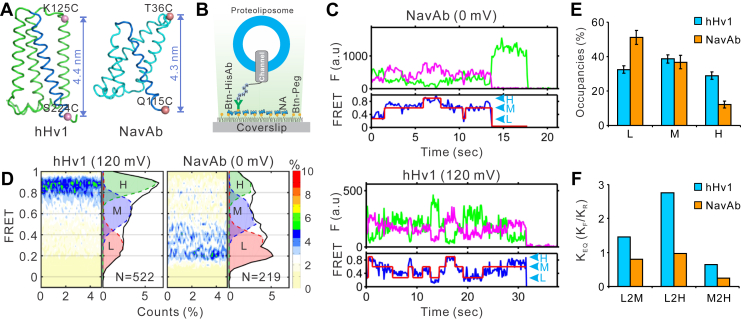
**Conformational dynamics of the NavAb voltage sensor revealed by smFRET.***A*, ribbon representation of the hHv1 and NavAb voltage sensors, with Cα carbons of K125/S224 sites in the hHv1 channel and T36/Q115 sites in the NavAb channel highlighted as *pink spheres*. S4 segments were colored *purple*. *B*, immobilizing hHv1 or NavAb proteoliposomes on PEG/PEG-biotin–coated coverslip surfaces for smFRET imaging with an objective-based TIRF microscope. The hHv1 or NavAb channel proteoliposomes were retained by biotinylated anti-Histag antibodies (Btn-HisAb), which bind to the coverslip surface coated with PEG and biotin-PEG (Btn-Peg) through neutravidin (N.A.). *C*, representative smFRET traces exhibited transitions among three FRET states collected from the T36/Q115 sites in the NavAb channel under 0 mV and the K125/S224 sites in the hHv1 channel under 120 mV. The liposome potential of 120 mV was generated by K^+^ gradient (In/Out =5/150 mM) in the presence of 0.45 μM valinomycin. The *purple* and *green* lines were donor and acceptor intensities, and the *blue* and *red* lines were real and idealized FRET, marked by *arrows* as high (H), medium (M), and low (L) FRET states. The smFRET traces were idealized as described in experimental procedures. *D*, the FRET histograms and contour maps from the 522 and 219 smFRET traces from the hHv1 and NavAb channels. Traces were extracted from over 10 smFRET movies collected from three independently prepared hHv1 or NavAb liposome samples. The high (*green*), medium (*purple*), and low (*red*) FRET populations were labeled as H, M, and L. *E* and *F*, FRET state occupancies and equilibrium constants collected from the K125C-S224C sites in the hHv1 channel and the T36C-Q115C in the NavAb channel. The state occupancies (*E*) were calculated from all idealized traces and presented as mean±s.e., n = 522 and 219 for hHv1 and NavAb, respectively. Equilibrium constants of transitions between low and medium (L2M), low and high (L2H), and medium and high (M2H) FRET states were calculated as ratios of the forward (K_F_) to reverse (K_R_) rates calculated from all smFRET traces. smFRET, single-molecule FRET.

In our previous work, we examined the conformational dynamics of the hHv1 channel using the smFRET approach. We introduced cysteine mutations, one pair at a time, at different sites in the hHv1 voltage sensor and examined the real-time FRET changes between the donor and acceptor fluorophores attached to these sites at a single channel level. Our results showed that the S4, not the S1-S3 segments, in the hHv1 voltage sensor exhibited transitions among three conformation states dependent on voltage, pH, and regulatory ligands, which were best reflected by FRET changes at the K125C-S224C sites (23, 24). However, the hHv1 channel is unique for only containing a voltage sensor without the separate pore-forming domain seen in other canonical VGICs (25). To further reveal the conformational landscapes of the voltage sensor in canonical VGICs, we introduced two cysteine mutations to the T36 and Q115 residues located at one of the four voltage sensors within the tetrameric NavAb channel unit (Fig. 3*A*). The T36C/Q115C-labeling sites are at locations equivalent to the K125C-S224C sites we previously used to monitor the conformational dynamics of the hHv1 S4 segment (Fig. 3*A*). The purified NavAb T36C/Q115C proteins were labeled with Cy3/Cy5 FRET fluorophore pair and then reconstituted into liposomes at an extremely low protein/lipid ratio of 1/4,000 (w/w), so the liposome populations without or with only one NavAb molecule were predominant. The proteoliposomes were immobilized on coverslip surfaces by anti-Histag antibodies. Thus, only liposomes with NavAb cytoplasmic 6xHistag facing outside were retained for smFRET imaging (Fig. 3*B*). Like what we observed on the hHv1 channel, the S4 helix of the NavAb channel also exhibited transitions among three major FRET states (Figs. 3*C* and S2). Hundreds of smFRET traces were collected from NavAb channels (V_1/2_ = −22 mV) under 0 mV and compared to those from the hHv1 channel (V_1/2_ = +10 mV) under 120 mV, so both were under fully activating voltages. Like hHv1 channels, smFRET traces from the NavAb channel were also heterogenous, with over 50% of traces exhibiting transitions among 2 to 3 states. The contour maps and histograms from the NavAb voltage sensor were also fitted well by three major FRET states with centers at 0.27, 0.6, and 0.9, like the hHv1 channel (Fig. 3*D*). However, the FRET distribution of the NavAb voltage sensor was very different from that of the hHv1 channel, with enriched low FRET-0.27 states and decreased high FRET-0.9 states (Fig. 3*E*). The FRET distribution of the NavAb channel is more like the hHv1 channel stabilized at the deep resting states by Zn^2+^, although it was under a strong activating voltage of 0 mV (23, 24). We further performed kinetic analyses using a kinetic model previously used for the hHv1 channel, which idealized all FRET traces with three FRET states with centers at 0.27, 0.6, and 0.9 (23, 24). Since smFRET measurements were performed on NavAb channels at equilibrium conditions, transition equilibrium constants are ratios of the forward to the backward transition rates. As shown in Figure 3*F*, the equilibrium constants of all transitional types, including low to medium FRET (L2M), low to high FRET (L2H), and medium to high (M2H), were all below 1, shifting the FRET distributions towards the low and medium FRET states, which is in sharp contrast to hHv1 channel under activating voltages.

## Discussion

Although the crystal structures of the NavAb channel were obtained over a decade ago, the function of purified NavAb proteins remained to be determined. Using liposome flux assay, we showed that NavAb channel proteins for structural studies were functional to conduct Na^+^. Moreover, our data showed that NavAb channels were also permeable to protons, confirming that the selectivity pore of the Nav channels is hydrated, therefore, able to conduct protons (4). In addition, we also demonstrated that lidocaine blocks the NavAb channel pore from conducting both Na^+^ and H^+^. Our functional characterizations provided direct experimental evidence to confirm that NavAb proteins used in many structural studies are functional.

As a canonical VGIC, the S4 segment in the NavAb voltage sensor contains four positively charged Arg residues to sense membrane voltage. Structures of the NavAb channel chemically ‘locked’ at resting and activated states have been obtained, which provided important insights into voltage sensing and gating mechanisms (15). However, real-time conformational transitions of voltage sensors in canonical VGICs remain unknown. Using the smFRET approach, our previous work on a standalone voltage sensor, the hHv1 channel, uncovered the conformational trajectories of the S4 segment, suggesting that it may travel over 20 Å in a lipid membrane environment (23). Our data met well with long-timescale molecular dynamics simulation results on the hHv1 channel and uncovered a new conformational population that may represent its real activated state (26). In the present work, we constructed a tandem tetrameric NavAb channel that allowed us to study its voltage sensor conformational dynamics using smFRET. We collected smFRET data from labeling sites in the NavAb voltage sensor equivalent to the hHv1 channel we studied previously, which indicated that the S4 segment of the NavAb voltage sensor also exhibits similar conformational landscapes. Moreover, its FRET histogram was fitted well with the same three FRET states used to fit that from the hHv1 channel in our previous work (23). These data suggested that although having a coupled pore domain, the NavAb S4 segment shares a conformational transition range similar to that of a standalone voltage sensor from the hHv1 channel.

Using the cryo-EM structures of the NavAb channel locked at resting and activating states, we simulated the accessible volumes of donor and acceptor fluorophore at the T36C- and Q115C-labeling sites using a FRET Positioning System (27), which then predicted the average FRET between them as 0.2 from the resting structure and 0.55 from the activated structure. We also calculated the average FRET between the K125C- and S224C-labeling sites in the hHv1 channel using structural models at the resting and the activated states provided by long-timescale molecular dynamics simulations (26), and the predicted FRET is 0.28 from the resting model and 0.57 from the activated model. The FRET states predicted by these structures agree well with two FRET populations with centers at 0.27 and 0.6, which appeared in our FRET histograms. Interestingly, all structures failed to predict the high FRET 0.9 population uncovered by our smFRET data, which is closely associated with voltage activation in the hHv1 channel in our previous work (23). Our smFRET results proposed that the S4 segment in the NavAb channel at the activated state captured by cryo-EM perhaps did not reach the most outward position, as suggested by electrophysiological studies (5, 17, 19).

Although both channels were studied under strong activating voltages, their conformational distributions are significantly different. The NavAb S4 segment appeared to adopt a conformational distribution similar to that of the hHv1 channel stabilized by Zn^2+^ at deep resting states (24). If only the high FRET conformations of the S4 segment are activated states, and all four voltage sensors are required to activate channel pore, the open probability with 12% high FRET state occupancy will be only 0.02%. Thus, to define the functional relevance of different conformational states, we need to determine the open probability of purified NavAb in giant liposomes and the conformational distributions of NavAb S4 segment at different voltages. Unfortunately, our smFRET measurements were performed only under 0 mV with symmetrical ionic/pH conditions. Since the NavAb channel conducts Na^+^, K^+^, and protons, under non-zero voltages, proton flows through NavAb channels will change the intraliposomal pH and equilibrium voltage. As a result, it is hard to determine changes in voltage sensor dynamics solely induced by voltage. Mutation abolishing ion conductance without altering voltage dependence needs to be identified and introduced into the NavAb channel to investigate how voltage modifies its voltage sensor dynamics in future studies.

## Experimental procedures

### Protein expression, purification, and fluorophore labeling

The NavAb cDNA carrying N49K mutation was kindly provided by Dr Katsumasa Irie of Nagoya University, Japan (28). The cDNA-encoding NavAb channel carrying N49K/T206A mutation was subcloned into the pET28 vector to express monomeric NavAb channels. The NavAb tandem tetramer was constructed by linking four NavAb cDNAs (N49K) with flexible linkers containing 2xGGGS and ‘LVPRGS’ thrombin-cutting sites between each subunit. The NavAb proteins were expressed in *E. coli* KRX host cells, and cell cultures were induced when A600 reached 0.6, by 0.1 mM isopropyl β-D-1-thiogalactopyranoside and 0.1% L-rhamnose (w/v) overnight under 25 °C. The cells were harvested and resuspended into the resuspension buffer containing 20 mM Tris–HCl, 150 mM NaCl, proteinase inhibitor cocktail (2 mM phenylmethylsulfonyl fluoride, 1 mM AEBSF, 1.5 μM Pepstatin, 1.4 μM E−64, 4 μM Bestatin), DNase I, pH 8.0, then homogenized by a Microfluidizer (Microfluidics Inc). The membranes were collected by ultracentrifugation under 4 °C, 100,000g for 3 h, then resuspended into the resuspension buffer with an additional 20 mM imidazole. The NavAb proteins were extracted from membranes by 1% (w/v) lauryl maltose neopentyl glycol (LMNG), under 4 °C for 3 h and then purified by Talon cobalt resin (Takara Inc) (23, 28). The affinity-purified NavAb proteins were further separated by a Superdex 200 size-exclusion column using the gel filtration buffer containing 20 mM Tris, 150 mM NaCl, 0.1% LMNG, pH 8.0. The tetramer fractions of NavAb proteins were pooled and concentrated by Amicon ultrafilters. The NavAb T36C/Q115C mutant proteins for smFRET studies were bound to Talon cobalt resin and then changed into the fluorophore-labeling buffer containing 20 mM Tris, 150 mM NaCl, 0.1% LMNG, pH 7.0. The NavAb proteins bound to cobalt resin were incubated with 100 μM Cy3/Cy5 c5 maleimide (1:1) with improved photostabilities (29) at 4 °C for 2 h. The free fluorophores were removed by the gel filtration buffer containing 20 mM imidazole and then eluted by the gel filtration buffer containing 500 mM imidazole. The fluorophore-labeled NavAb tetramer proteins were further separated by a Superdex 200 column and then either reconstituted immediately or stored in a −80 °C freezer. The human voltage-gated proton channel hHv1 was expressed, purified, labeled, and reconstituted as described previously (23, 24).

### Liposome reconstitution

The 1-palmitoyl-2-oleoyl-sn-glycero-3-phosphoethanolamine/1-palmitoyl-2-oleoyl-sn-glycero-3-phospho-(1'-rac-glycerol) (3:1, w/w, Avanti Polar Lipids Inc) lipids dissolved in chloroform were dried in clean glass tubes using argon and then placed in a vacuum desiccator for 4 h to remove the organic solvents completely. The dried lipids were resuspended in the reconstitution buffer containing 20 mM Hepes, 150 mM KCl, pH 7.5. The liposomes were sonicated and then solubilized with 1% (w/v) LMNG (Anatrace Inc). The purified NavAb proteins were mixed with lipids at a ratio of 1:200 (w/w) for liposome flux assay, 1:4,000 (w/w) for smFRET imaging, and NavAb proteoliposomes were formed by detergent removal using Bio-beads SM2 (Bio-Rad Inc) under 4 °C overnight. The NavAb proteoliposomes were immediately used or stored in a −80 °C freezer.

### Liposome flux assay

The K^+^ gradient across liposomes is established by diluting the liposomes in the extraliposomal buffer containing 20 mM Hepes, 150 mM NMDG, pH 7.5, for 20×. The liposomes were incubated with 2 μM of fluorophore ACMA for ∼5 min. Initial ACMA fluorescence intensities (F_0_) were measured for ∼5 min using a FluoStar plate reader with excitation and emission wavelength setting to 390 and 460 nm. The ACMA fluorescence intensities were continuously monitored after adding 0.45 μM K^+^ ionophore valinomycin. The NavAb channel activities were calculated from the normalized fluorescence readings using the following equation:

$$Activities=\left(F_{0}-F_{val}\right)/F_{0}$$

where F_0_ and F_val_ are the steady state ACMA fluorescence intensities before and after adding 0.45 μM valinomycin.

### smFRET imaging and data analysis

The NavAb and hHv1 proteoliposomes were immobilized by biotinylated anti-Histag antibodies (Thermo Fisher Scientific, MA1-21315-BTIN) attached to PEG/biotin-PEG–passivated coverslip surfaces prepared following the protocol of Joo *et al* (30). SmFRET imaging was performed with an objective-based TIRF, and liposome voltage applied to hHv1 channels were generated by transliposomal K^+^ gradients in the presence of 0.45 μM valinomycin as described previously (23, 24). A 532 nm laser (∼1.0 W/cm2) was used to excite donor fluorophores, and a minimum of three batches of smFRET movies were collected for each sample/condition at 10 frames per second. All imaging buffers contained ∼3 mM Trolox, 5 mM protocatechuic acid, and 15 μg/μl of protocatechuate-3,4-dioxygenase to enhance the photostability of the fluorophores (31). Raw smFRET movies without any corrections were processed by the SPARTAN software (https://www.scottcblanchardlab.com/software) (32) with the point spread function window size as seven pixels, and smFRET traces were selected using the Autotrace function and further manually inspected following criteria described previously (23, 24). The kinetic analyses were conducted on all smFRET traces with a four FRET state model, and the additional 0 FRET state was introduced in kinetic analysis to minimize the impacts of blinking and bleaching FRET events. The smFRET traces were idealized using the Maximum Point Likelihood algorithm built into the SPARTAN software (32, 33).

## Data availability

All data are contained within the article.

## Supporting information

This article contains supporting information.

## Conflict of interest

The authors declare that they have no conflicts of interest with the contents of this article.
