# Supplementary material for: Voltage sensor dynamics of a bacterial voltage-gated sodium channel NavAb reveal three conformational states
Source: J Biol Chem. 2023 Feb 1;299(3):102967. doi: 10.1016/j.jbc.2023.102967 (PMC9986516; doi:10.1016/j.jbc.2023.102967)
Supplement: Supplemental data [file mmc1.pdf]

Voltage sensor dynamics of a bacterial voltage-gated sodium channel NavAb reveal three conformational states

Shuo Han[#], Joshua Vance[#], Samuel Jones, Jenna DeCata, Kimberly Tran, John Cummings and Shizhen Wang[*]

Division of Biological and Biomedical Systems

School of Science and Engineering

University of Missouri-Kansas City, Kansas City, MO 64110 USA

*Corresponding to S.W. (wangshizhen@umkc.edu)

[#]Authors contributed equally to this work

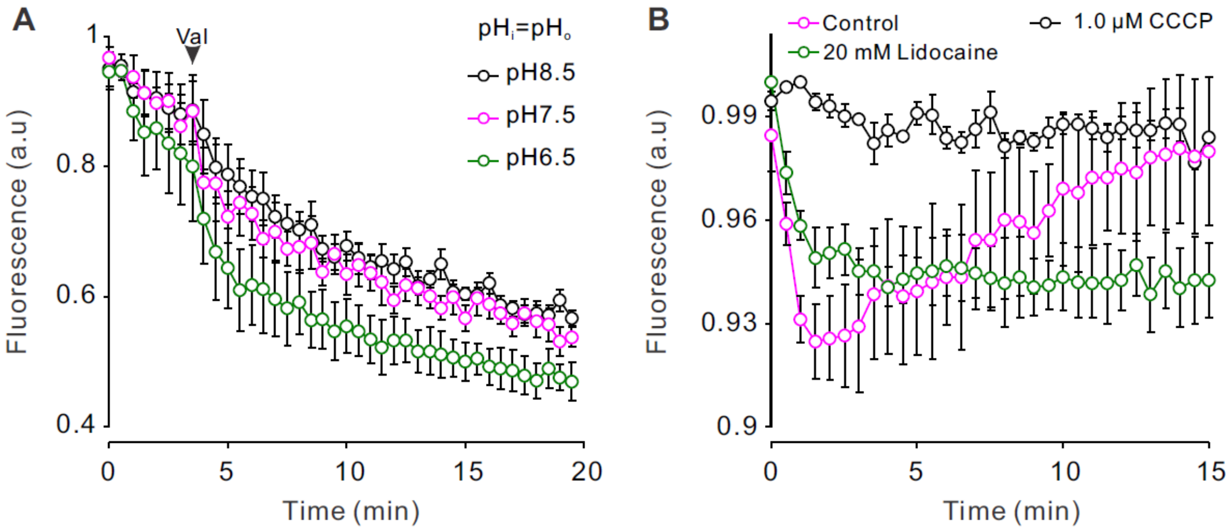

Fig S1. Liposome flux assay of NavAb channels reconstituted into liposomes at different pH (A) or with proton gradient (B)

A. The NavAb channels were reconstituted into liposomes with different pH at the intra- (pHi) and extraliposomal (pHo) sides. The liposome flux assays were performed as described in experimental procedures. Lower intra- and extra-liposomal pH, driven by the same K^+ gradients, led to more acidic accumulation, therefore, slightly more ACMA fluorescence quenching. Data were presented as mean±s.e., n=6. **B**. The NavAb channels were reconstituted into liposomes with intraliposomal pH of 6.5 and extraliposomal pH of 8.5. The ACMA (20 µL) was preincubated with NavAb liposomes and injected into the extraliposomal buffer (20 µL) to trigger the assay. The initial ACMA quenching by NavAb liposomes with lower intraliposomal pH was recovered slowly due to dissipating the H^+ gradient by NavAb channels, which were sensitive to lidocaine block. In the presence of 1 µM proton ionophore CCCP, the dissipation of the H^+ gradient was too fast to observe the initial ACMA quenching. Data were presented as mean±s.e., n=3.

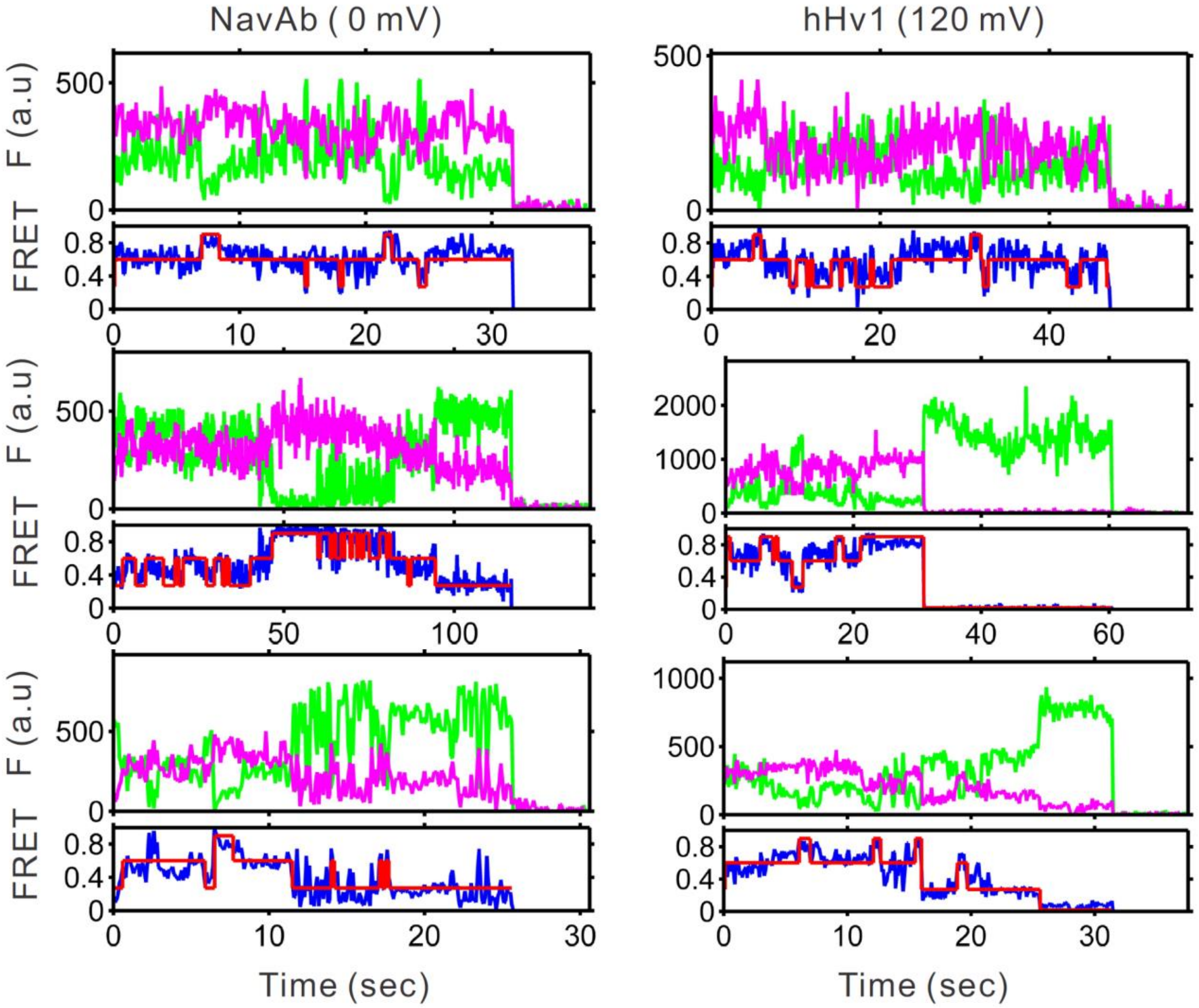

Fig S2. Representative smFRET traces exhibiting transitions among 3 FRET states from the T36/Q115 sites in the NavAb channel under 0 mV and the K125/S224 sites in the hHv1 channel under 120 mV

The liposome potential of 120 mV was generated by K^+ gradient (In/Out =5/150 mM) in the presence of 0.45 μM valinomycin. The purple and green lines were donor and acceptor intensities, and the blue and red lines were real and idealized FRET.
